# Fiber Raman Amplifiers and Fiber Raman Lasers

**DOI:** 10.3390/mi11121044

**Published:** 2020-11-27

**Authors:** Luigi Sirleto

**Affiliations:** Institute of Applied Sciences and Intelligent Systems (ISASI), CNR, 80131 Napoli, Italy; luigi.sirleto@cnr.it

Stimulated Raman scattering (SRS) is a nonlinear optical effect, observed for the first time in 1962, which lies at the heart of fiber Raman amplifiers and fiber Raman lasers. SRS can be obtained by irradiating a sample with two simultaneous light sources: a light wave at frequency *ω_L_* (the pump laser wave) and a light wave at frequency *ω_S_* = *ω_L_* − *ω_υ_* (the Stokes Raman wave), where *ħω_υ_* corresponds to a vibrational energy. When the frequency difference between the pump and the Stokes laser beams matches a given molecular vibrational frequency of the sample under test, there is a transfer of energy from the high power pump beam to the probe beam, which can be co-propagating or counter-propagating. The SRS effect occurs in the form of a gain for the Stokes beam power. In SRS, many Stokes frequencies can be generated at the output when the pump power exceeds the threshold value, thus Stokes frequencies at *ω_P_ − ω_ν_*, *ω_P_ −* 2*ω_ν_* can be observed, leading to the so-called “cascaded” SRS [1]. In the last two decades, a renewed interest has been addressed to SRS due to interesting applications in nano [2,3,4] and biophotonics [5,6]. However, in the field in which SRS manifests, at the best, its potentiality is fiber optics [7,8,9,10]. Fiber optics amplifiers and lasers based on SRS are related to basic physics aspects and a vast number of applications, at the same time. 

In fiber Raman amplifiers (FRAs), a power transfer from pump to information carrying the signal (usually described as a probe) can occur if there is a sufficient pump power within the fiber. In principle, when optical pump energy is added, along with signals in ordinary optical fibers, optical amplification can take place, providing low-noise, flat, and wideband signal gain. The fundamental advantages of FRAs are: (1) they do not require special dopants. Raman gain is obtainable in any conventional transmission fiber, which can be used as a transmission line and as a Raman gain medium, too. Therefore, Raman amplification is compatible with most available transmission systems. (2) Raman amplification can be provided at any wavelength. Being the Raman gain non-resonant, it is available over the entire transparency region of fiber, if the appropriate pump sources are available. (3) Raman gain has a high-speed response. (4) FRAs, operating in a signal band outside the erbium-doped fiber amplifier (EDFA) bands, could open new transmission windows in the future [7,8,9,10]. In the past century, fused silica has been the main material used for long and short-haul transmission of optical signals, because of its good optical properties and attractive figure of merit (trade-off between Raman gain and losses). Nowadays, an interesting aspect is the development of glasses with a higher Raman gain coefficient and with an increased Raman gain bandwidth [11,12]. 

Raman amplifiers are generally divided into two categories, namely, distributed and lumped amplifiers. In a distributed FRA, the pump at the optical frequency *ω_P_* and signal at the frequency *ω_s_* are jointly launched into the same ordinary optical fiber. Raman optical amplification takes place when their frequency difference Ω = *ω_P_* − *ω_s_* lies within the bandwidth of the fiber Raman-gain spectrum. The distributed FRA guides light at both the signal and pump wavelengths, and it is normally in single mode to ensure the best overlap of all traveling waves. Distributed FRAs offer several advantages: simplicity, because they provide direct optical amplification of signal light in the transmission fiber; flexibility in the use of signal wavelengths, because the Raman gain peak is dependent on the pump wavelength and not the emission cross section of a dopant; and broad gain bandwidth, which allows to employ multiple pumps. The major benefits, only obtainable through the use of distributed amplification, is that the signal gain may be pushed into a transmission span preventing the signal from reduction in power. Thus, lower signal powers can be used, nonlinear penalties are reduced, and higher loss can be tolerated [13,14]. Lumped or discrete FRAs use a fiber medium that is localized before or after transmission to fully or partially compensate for the transmission loss. Discrete FRAs are primarily used to increase the capacity of fiber-optic networks, opening up new wavelength windows, which are inaccessible by EDFAs. In FRAs, the signal is amplified at a wavelegth given by the frequency difference between the pump and the Stokes frequencies, thus, by choosing the pump wavelength, the gain at any wavelength can be obtained. Moreover, by combining multiple pump wavelengths centered at different wavelengths, a flat gain in an ultra wide bandwidth is achievable [15]. Dispersion-compensating Raman amplifiers are interesting because they integrate two crucial tasks, dispersion compensation and discrete Raman amplification, into a single component [16].

High-power fiber lasers have achieved output powers of multiple kilowatts from a single fiber. Due to its inherent material advantages, Ytterbium has been the primary rare-earth-doped gain medium, so fiber lasers are largely confined to its narrow emission wavelength region. Fiber Raman lasers (FRLs) can lead to conversion to wavelengths higher than the starting wavelength, generating several Stokes orders by cascading effects. The most important advantage of Raman lasers is that any laser wavelength can be achieved from the ultraviolet to the infrared with a suitable choice of the pump wavelength, providing that wavelengths are within the transparency region of the material and sufficiently high nonlinearity and/or optical intensity are reached. For this reason, currently, FRLs are the only wavelengths scalable, high-power fiber laser technology that can span the wavelength spectrum [17]. Fiber Raman lasers (FRLs) are similar to ordinary lasers. A first analogy is that, in FRLs, lasing occurs when the Raman-active gain medium is placed inside a cavity, for example, between mirrors reflecting the first Stokes wavelength. A second analogy is that, in FRLs, the threshold power is obtained when Raman amplification during a round trip is as large as compensating the cavity losses. However, there are also some important differences between FRLs and traditional lasers. The first difference is that an amplifier medium based on Raman gain is used rather than on stimulated emission from excited atoms or ions. The second difference is that the required wavelength for pumping Raman lasers does not depend on the electronic structure of the medium, so it can be chosen to minimize absorption. 

Raman lasers in fiber can be classified into two general categories. In the former, the wavelength is shifted by one Raman–Stokes shift, while, in the latter, called the cascaded Raman laser, the wavelength is shifted by multiple Raman–Stokes shifts. A single wavelength shift FRL is a fiber resonator at the Stokes wavelength, in which SRS shifts the spectrum of the propagating pump radiation through an optical fiber towards lower frequency Stokes components. Raman lasing is obtainable in conventional single-mode telecom fibers, as well as other passive fibers, by trapping Stokes components by reflectors and by pumping the laser by a high-power rare-earth-doped fiber laser. A multiple wavelength shifts FRL takes advantage of SRS cascading. The pump light gives rise to the “first-order” laser light in a single frequency-shifting step, which remains trapped in the laser resonator. Afterwards, the “first-order” laser light can be pushed to very high power levels, becoming itself the pump for the generation of the “second-order” laser light, which is shifted by the same vibrational frequency of the first order. Using this technique, conversion of the pump light to an “arbitrary” desired output wavelength through several discrete steps can be performed by using a single laser resonator.

Tunability is an important property for lasers. In order to obtain a tunable FRL, cavity mirrors can be integrated within the fiber by tunable fiber Bragg gratings [18]. Germanosilicate fibers are extensively used in FRLs because their Raman gain value is about eight times higher than silicate fiber. In a GeO–doped fiber, taking advantage of a linear cavity configuration and a purely axial compression of the fiber bragg gratings (FBGs), a high-power and widely tunable all-fiber Raman laser can be obtained with continuously tuning over 60 nm [19]. Low-loss phosphosilicate (P_2_O_5_–SiO_2_) fiber has a peak Raman shift of 1330 cm^−1^, therefore to make a FRL at 1484 nm, only two cascaded cavities (at 1239 nm and 1484 nm, respectively) are required, thereby greatly increasing the FRL efficiency. In addition, in phosphosilicate (P_2_O_5_–SiO_2_) fiber, a FRL with a tuning range of about 50 nm can be obtained [20]. 

For applications requiring high power laser with wavelengths longer than 2 microns, FRLs are an attractive option [21]. Chalcogenide glass fibers have Raman gain coefficients approximately two orders of magnitude greater than the gain coefficients of silica. For wavelengths longer than 1.8 µm, the development of Raman fiber lasers based on chalcogenide glasses has now become technically achievable [22].

Single-frequency laser sources are difficult to implement, because they suffer from poor robustness and quality (linewidth and stability) and are expensive. A Raman gain based on a distributed feedback (DFB) fiber laser has a number of potential advantages: first, the possibility of generating narrow linewidth low-noise oscillation in wavelength bands outside the band of rare-earth doped materials; second, Raman-based fiber laser systems do not suffer from issues associated with high-concentration rare-earth doped fibers, which limit their efficiency due to thermal effects [23,24].

By exploiting the multiple scattering of photons in a disordered gain medium, a random laser can be obtained, allowing a coherent light source without a traditional cavity. For different scientific and medical applications, ultrafast Raman fiber lasers are an interesting option. An efficient way to obtain a high power ultrafast Raman fiber laser is pulse pumping, but it requires a real-time synchronization between the pump pulses and laser cavity. In order to overcome this limitation, random fiber lasers with distributed Raman gain and Rayleigh feedback in standard telecommunication optical fibers have been realsed. Their advantages are a simple and flexible design, quasi-CW operation, narrow spectrum generation, high beam quality, and pump energy conversion efficiencies comparable to the efficiencies of conventional cavity lasers [25] and wavelength tuning [26]. 

Optical communication systems are often limited by fiber dispersion, which broadens the pulse by fiber losses. In principle, the use of fundamental solitons, as an information bit, can solve the prolem of dispersion [27]. However, due to the fiber losses, soliton width begins to increase because of a decrease in the peak power during propagation inside the fiber; therefore, amplification is required in order to recover original width and peak power. Taking advantage of SRS, solitons can be amplified by injecting CW pump radiation periodically [28]. 

When the wavelength of the pump pulse is close to or inside the anomalous dispersion region of an optical fiber, the Raman pulse should experience the soliton effects, i.e., under suitable conditions, almost all of the pump-pulse energy can be transferred to a Raman pulse that propagates undistorted as a fundamental soliton. Raman solitons were also generated by using a conventional fiber with the zero-dispersion wavelength near 1.3 µm, which led to 100-fs Raman pulses near 1.4 µm [29]. An interesting application of the soliton effects led to the development of the Raman soliton laser. Such lasers provide their output in the form of solitons of widths 100 fs, but at a wavelength corresponding to the first-order Stokes wavelength, which can be tuned over a considerable range [30]. 

In fiber optic communications, the growing demand in terms of transmission capacity has fulfilled the entire spectral band of the erbium-doped fiber amplifiers (EDFAs). This dramatic increase in bandwidth rules out the use of EDFAs, leaving fiber Raman amplifiers (FRAs) as the key devices for future amplification requirements. Today, almost every long-haul or ultralong-haul fiber-optic transmission system uses FRAs. In the field of high-power fiber lasers, commercially available fiber-based Raman lasers can deliver output powers in the range of a few tens of Watts in continuous-wave operation with high efficiency and broad gain bandwidth, covering almost the entire near-infrared region. Due to their tunability, compactness, and capability for multi-wavelength operation, FRLs have great commercial potential in a variety of applications. 

However, for both FRAs and FRLs, a number of interesting basic physical challenges still remain open and a number of new applications could be envisaged for the future implementation of high-capacity optical communication systems and for the future realization of high power fiber lasers, respectively.

## References

[1] Shen Y.R., Bloembergen N. (1965). Theory of stimulated Brillouin and Raman scattering. Phys. Rev..

[2] Sirleto L., Ferrara M.A., Nikitin T., Novikov S., Khriachtchev L. (2012). Giant Raman gain in silicon nanocrystals. Nat. Commun..

[3] Sirleto L., Vergara A., Ferrara M.A. (2017). Advances in stimulated Raman scattering in nanostructures. Adv. Opt. Photonics.

[4] Ferrara M.A., Sirleto L. (2020). Integrated Raman Laser: A Review of the Last Two Decades. Micromachines.

[5] Camp C.H., Cicerone M.T. (2015). Chemically sensitive bioimaging with coherent Raman scattering. Nat. Photonics.

[6] Ferrara M.A., Filograna A., Ranjan R., Corda D., Valente C., Sirleto L. (2019). Three-dimensional label-free imaging throughout adipocyte differentiation by stimulated Raman microscopy. PLOS ONE.

[7] Agrawal G. (2012). Nonlinear Fiber Optics.

[8] Agrawal G.P. (2011). Nonlinear fiber optics: Its history and recent progress. J. Opt. Soc. Am. B.

[9] Headley C., Agrawal G.P. (2004). Raman Amplification in Fiber Optical Communication Systems.

[10] Sirleto L., Ferrara M.A. (2020). Fiber Amplifiers and Fiber Lasers Based on Stimulated Raman Scattering: A Review. Micromachines.

[11] Pernice P., Sirleto L., Vergara A., Aronne A., Gagliardi M., Fanelli E., Righini G.C. (2011). Large Raman Gain in a Stable Nanocomposite Based on Niobiosilicate Glass. J. Phys. Chem. C.

[12] Sirleto L., Aronne A., Gioffrè M., Fanelli E., Righini G.C., Pernice P., Vergara A. (2013). Compositional and thermal treatment effects on Raman gain and bandwidth in nanostructured silica based glasses. Opt. Mater..

[13] Islam M.N. (2003). Raman Amplifiers for Telecommunications 1.

[14] Pelouch W.S. (2016). Raman Amplification: An Enabling Technology for Long-Haul Coherent Transmission Systems. J. Lightwave Technol..

[15] Kobtsev S.M., Pustovskikh A.A. (2004). Improvement of Raman Amplifier Gain Flatness by Broadband Pumping Sources. Laser Phys..

[16] Lewis S.A.E., Chrenikov S.V., Taylor J.R. (2000). Broadband high-gain dispersion compensating Raman amplifier. Electron. Lett..

[17] Supradeepa V.R., Feng Y., Nicholson J.W. (2017). Raman fiber lasers. J. Opt..

[18] Bélanger E., Déry B., Bernier M., Bérubé J.P., Vallée R. (2007). Long term stable device for tuning fiber Bragg gratings. Appl. Opt..

[19] Bélanger E., Bernier M., Faucher D., Côté D., Vallée R. (2008). High-Power and Widely Tunable All-Fiber Raman Laser. J. Lightwave Technol..

[20] Babin S.A., Churkin D.V., Kablukov S.I., Rybakov M.A., Vlasov A.A. (2007). All-fiber widely tunable Raman fiber laser with controlled output spectrum. Opt. Express.

[21] Dianov E.M., Prokhorov A.M. (2000). Medium-power CW Raman fiber lasers. IEEE J. Sel. Top. Quantum Electron..

[22] Thielen P.A., Shaw L.B., Pureza P.C., Nguyen V.Q., Sanghera J.S., Aggarwal I.D. (2003). Small-core As–Se fiber for Raman amplification. Opt. Lett..

[23] Shi J., Alam S., Ibsen M. (2012). Highly efficient Raman distributed feedback fiber lasers. Opt. Express.

[24] Loranger S., Karpov V., Schinn G.W., Kashyap R. (2017). Single frequency low threshold linearly polarized DFB Raman fiber lasers. Opt. Lett..

[25] Churkin D.V., Kolokolov V., Podivilov E.V., Vatnik I.D., Nikulin M.A., Vergeles S.S., Terekhov I.S., Lebedev V.V., Falkovich G., Babin S.A. (2015). Wave kinetics of random fiber lasers. Nat. Commun..

[26] Babin S.A., El-Taher A.E., Harper P., Podivilov E.V., Turitsyn S.K. (2011). Tunable random fiber laser. Phys. Rev. A.

[27] Haus H.A., Wong W.S. (1996). Solitons in optical communications. Rev. Mod. Phys..

[28] Molleanauer L.F., Smith K. (1988). Demonstration of soliton transmission over more than 4000 km in fiber with loss periodically compensated by Raman gain. Opt. Lett..

[29] Gouveia-Neto A.S., Gomes A.S.L., Taylor J.R. (1987). High-efficiency single-pass solitonlike compression of Raman radiation in an optical fiber around 1.4 µm. Opt. Lett..

[30] Ding M., Kikuchi K. (1994). Improvement of the fiber raman soliton laser for femtosecond optical pulse generation. Fiber Integr. Opt..

